# Efficacy of keratinocyte growth factor (palifermin) for the treatment of caustic esophageal burns

**DOI:** 10.3892/etm.2014.1851

**Published:** 2014-07-17

**Authors:** KEMAL VARIM NUMANOĞLU, DUYGU TATLI, SIBEL BEKTAŞ, EBUBEKIR ER

**Affiliations:** 1 Department of Pediatric Surgery, Faculty of Medicine, Bülent Ecevit University, Kozlu, Zonguldak 67600, Turkey; 2 Department of Pathology, Faculty of Medicine, Bülent Ecevit University, Kozlu, Zonguldak 67600, Turkey

**Keywords:** keratinocyte growth factor, caustic burn, esophagus

## Abstract

Current treatment strategies against the development of corrosive esophageal strictures remain unsatisfactory. Thus, the aim of the present study was to investigate the efficacy of keratinocyte growth factor, in the form of palifermin, for the prevention of stricture development following esophageal caustic injuries in a rat model. A total of 32 female Wistar albino rats were divided into four groups, which included the control (C), burn (B), steroid (S) and steroid plus palifermin (S/P) groups. An experimental corrosive esophageal burn model was established in the B, S and S/P groups. Weight gain was recorded and histopathological evaluation was performed for each group. Weight gain in the S and B groups was compared with the control group and statistically significant differences were observed. In addition, statistically significant differences in weight gain were observed between the S/P group and the B group. Histopathologically, statistically significant differences were identified with regard to submucosal collagen deposition, muscularis mucosa and tunica muscularis damage when comparing the B group with the C group. In addition, statistically significant differences were observed when comparing the S and S/P groups with the B group. Furthermore, significant submucosal collagen deposition and tunica muscularis damage were observed in the S group when compared with the S/P group. The stenosis indexes in the C and S groups were significantly lower compared with the B group. In addition, the stenosis index in the S/P group was significantly lower compared with the S group. To the best of our knowledge, the present study is the first to investigate the effect of palifermin on corrosive esophageal burns. The addition of palifermin to the corrosive esophageal burn standard treatment regimen was found to reduce the degree of fibrosis and ameliorate histopathological damage in an experimental model of corrosive esophagitis in rats.

## Introduction

Corrosive substances are commonly used in daily life, and accidents due to these substances continue to be a frequent reason for the emergency hospital admission of children throughout the world. This hazard remains despite aggressive accident prevention education programs aimed at children and adults, preventive labeling and packaging and even legislation limiting the strength and availability of caustic substances. In rural areas and in developing countries, such as Turkey, caustic soda in crystal and liquid form is used in home industries for soap making, fruit drying and cleaning containers on farms. In addition, the high number of over-the-counter caustic cleaning agents means that children are likely to continue to accidentally ingest these agents. The natural curiosity of children and their tendency to taste everything, coupled with the availability of certain chemicals around the house, create the setting for corrosive esophageal injury. Although the mortality rate is not high, accidental ingestion of corrosive substances is usually harmful and results in lifelong damage (1). It is reported that 20–40% of the cases with corrosive substance ingestion result in esophageal damage (2,3). The main strategy for corrosive esophageal burn treatment involves the inhibition of inflammation, bacterial colonization and the subsequent narrowing that may occur (4,5). When a corrosive agent has been ingested, the presence or absence of an esophageal burn must be established immediately, and if a burn is diagnosed, the aim is to prevent stricture formation. Treatment methods for the prevention of stricture formation include antibiotics and steroids alone or in combination with bouginage, total parenteral nutrition, nasogastric tube and intraluminal stents. However, in previously reported studies, the stricture ratios have remained high (3,4). None of the treatment strategies have yielded uniform success, and they have failed to receive universal acceptance. This has resulted in an increasing number of studies investigating novel therapeutic strategies for the prevention of stricture formation in the esophagus following burns. However, despite experimental evidence indicating that the majority of agents (heparin, pentoxifylline, estradiol, antioxidants and epidermal growth factor) are beneficial in preventing esophageal strictures, the agents have not yet gained clinical application, possibly due to inconsistencies resulting from the requirement for systemic administration of these drugs. In addition, a number of the drugs have not yet been shown to be suitable for human use (6). Therefore, further investigation is required to determine the efficacy of different treatments in eliminating stricture formation.

Keratinocyte growth factor (KGF), a member of the growth factor family, exerts its effect by inducing epithelial proliferation, migration, and in certain tissues, epithelial differentiation. KGF suppresses inflammation by modulating the cytokine profile. The growth factor induces the upregulation of enzyme expression, which detoxifies free oxygen radicals and exerts a cytoprotective effect by inhibiting DNA breakage and epithelial cell apoptosis. KGF has a trophic effect in the oral and intestinal mucosa and is able to prevent atrophic alterations in these tissues (7,8).

Palifermin (Kepivance^®^) is a recombinant form of human KGF produced in *Escherichia coli*. In palifermin, the first 23 N-terminal amino acids found in endogenous KGF were deleted in order to obtain a more stable protein product. Palifermin was approved by the US Food and Drug Administration (FDA) in December 2004 to decrease the incidence and duration of severe oral mucositis in patients with hematological malignancies receiving myelotoxic therapy and requiring hematopoietic stem cell support. Although the FDA has only approved palifermin for use in bone marrow transplantation in the treatment of hematological malignancies, it is currently under investigation for use in additional therapies targeting mucosal injury, including colorectal cancer chemotherapy regimens and head and neck radiation (9). In the present study, the therapeutic effect of palifermin for the prevention of esophageal strictures was investigated in a rat alkaline esophageal injury model.

## Materials and methods

### Ethics

Experimental protocols were approved by the Experimental Ethics Committee of Bülent Ecevit University School of Medicine (Kozlu, Turkey). All the rats were housed at 24±1°C under controlled lighting (12-h light/dark cycle), humidity and human activity. Animals were allowed to acclimatize to these conditions for 10 days prior to the start of the experiment. All the study groups rats were housed in identical wire-bottomed cages to prevent coprophagy, and all animals received human care. Following ethical approval, a preliminary study was performed to standardize the experimental model and surgical techniques.

### Experimental model

A total of 32 female Wistar albino rats, weighing 190–240 g, were divided into four groups, which included the control (C), burn (B), steroid (S) and steroid plus palifermin (S/P) groups. An experimental corrosive esophageal burn model was established in the B, S and S/P groups.

Following overnight fasting, rats were anesthetized with 10 mg/kg xylazine (2%) (Rompun^®^, Bayer Healthcare AG, Leverkusen, Germany) and 100 mg/kg ketamine (Ketalar^®^, Pfizer, New York, USA) subcutaneously. The experimental model of caustic esophageal burn was established as previously described by Gehanno and Guedon (10), with modifications as in the study by Liu and Richardson (11). Following a median laparotomy, a 2-cm segment of the abdominal esophagus at the gastroesophageal junction was dissected and isolated. A catheter (1 mm internal diameter and 2.2 mm external diameter) (Bıçakçılar, Istanbul, Turkey) was advanced to the upper region of the isolated segment, while a venous catheter of 24 G size was introduced into the lower part of the segment via the stomach for drainage. The two ends of the segments were then secured. A total of 10 ml sodium hydroxide at 30% concentration was infused through the upper catheter for 90 sec in the B, S and S/P groups, whilst isotonic saline was used in the control group. Subsequently, distilled water was used to irrigate the burned segment for ≥15 sec. The catheters were then withdrawn and the gastric insertion site was repaired. Following closure of the laparotomy, 10 ml saline and 5% dextrose solutions (İE Ulagay, Istanbul, Turkey) were injected intraperitoneally. The rats were subsequently fasted for 24 h following surgery. At postoperative day one, intraperitoneal injections of the study group’s medications were initiated and rats were provided with food *ad libitum*. All the animals were housed in identical cages that provided food and water during the study period.

### Study design

In the control group, the esophagus of the rats was uninjured and untreated. For the rats in group B, a standard esophageal burn was produced, and the group received intraperitoneal injections of 150 mg/kg/day ampicillin (Ampisina^®^; Mustafa Nevzat Pharmaceuticals, Istanbul, Turkey). With regard to the rats in group S, the esophagus was injured and treated with 150 mg/kg/day ampicillin and 1 mg/kg/day dexametazon (Dekort^®^; Deva, Istanbul, Turkey) intraperitoneally. In the S/P group, the esophagus was injured and the rats were intraperitoneally administered 150 mg/kg/day ampicillin, 1 mg/kg/day dexametazon and 60 μg/kg/day palifermin (Kepivance^®^; Amgen, Thousand Oaks, CA, USA). The drugs were administered daily throughout the seven day treatment period. During the observation period, animals were weighed daily. The rats were sacrificed after 21 days. The efficacy of the treatment was subsequently assessed by analyzing the weight gain, stenosis index and histopathological evaluation of the burned segments in the rats in each group.

At postoperative day 21, the animals were sacrificed and the esophageal burn segment was removed, fixed in 10% formaldehyde and embedded in paraffin. Next, 5-μm sections were stained with hematoxylin-eosin and Masson’s trichrome connective tissue dye (Merck Millipore, Billerica, MA, USA). Esophageal wall thickness and luminal diameters were assessed using a millimetric ocular microscope. The thickness of the esophageal wall and the lumen diameter were measured in order to calculate the stenosis index (12). The stenosis index was calculated as follows: Stenosis index = [wall thickness (A1 + A2)/2]/[lumen diameter (B1 + B2)/2]. Submucosal collagen accumulation, muscularis mucosa injury, tunica muscularis injury and collagen accumulation were evaluated semi-quantitatively and scored as shown in Table I (1).

### Statistical analysis

Mean values with the standard deviation of the body weights in each group were calculated prior to and following the experiment. The differences between the body weights prior to and following the experiment were analyzed using Kruskal-Wallis variance analysis, while pairwise comparisons between the groups were performed using the Bonferonni-corrected Mann-Whitney U-test. Histopathological scores were analyzed using the χ^2^ and Fisher’s exact tests. P<0.05 was considered to indicate a statistically significant difference. Statistical calculations were performed using SPSS statistical software, version 13 (SPSS Inc., Chicago, IL, USA).

## Results

### Mortality and body weight

Two rats were excluded from the study following mortality on day 6 and day 13. The body weights of all the animals were measured prior to and at the end of the study (day 21; Table II). A statistically significant difference was observed in the body weights between the B group and the C group (P<0.05). In addition, a statistically significant difference was observed with regard to the body weight between the S/P-treated group and the B group (P=0.012).

### Histopathological observations

**Figure 1:**
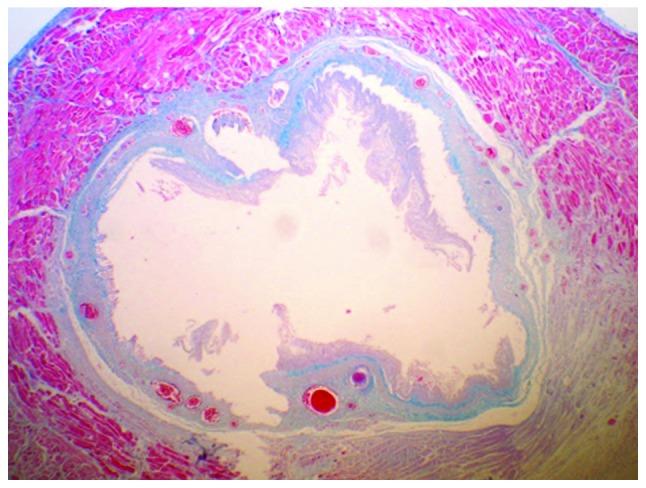
Widespread collagen accumulation was observed in the submucosal and muscular layers in the burn group (magnification, ×100; staining, hematoxylin-eosin and Masson’s trichrome).

**Figure 2:**
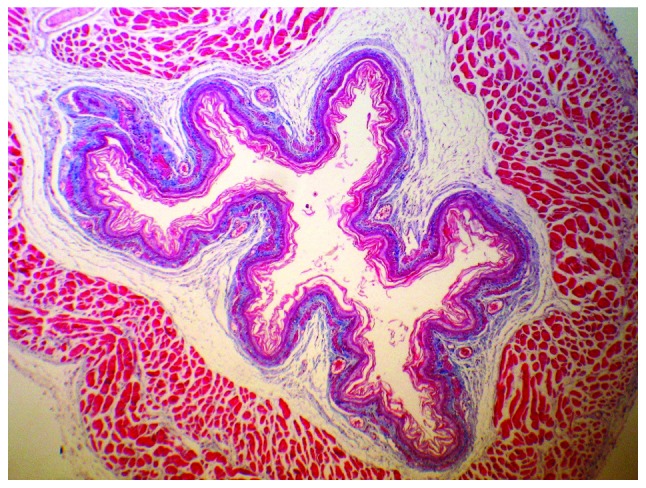
A mild increase in collagen deposition was observed in the muscularis mucosa, submucosa and tunica muscularis in the steroid treatment group (magnification, ×100; staining, hematoxylin-eosin and Masson’s trichrome).

Esophageal sections were prepared from all the rats and were evaluated for submucosal collagen accumulation, damage to the muscularis mucosa, damage to the tunica muscularis and collagen accumulation. The corrosive burn group was examined and prominent damage in the mucosal, submucosal and muscular layers was observed. In addition, a widespread accumulation of collagen in the submucosal and muscular layers was identified (Fig. 1). A mild thickening of the muscularis mucosa was observed during the histopathological examination of the steroid-treated group. Furthermore, a mild increase in collagen deposition was observed in the muscularis mucosa, submucosa and the tunica muscularis of the S group when compared with the B group (Fig. 2).

**Figure 3:**
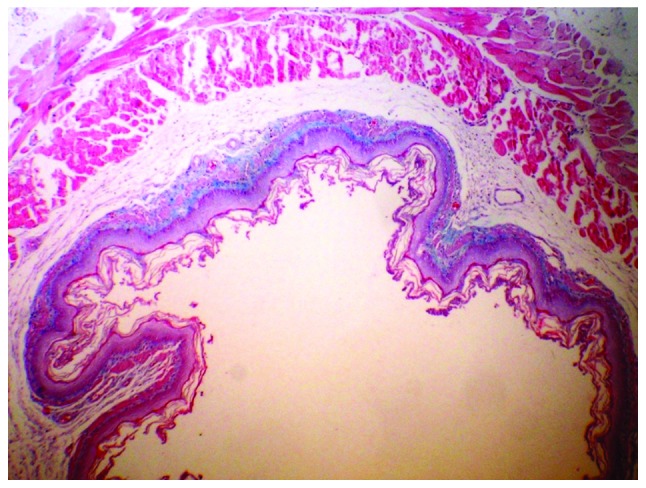
Regular mucosal, submucosal and muscular structures were observed in the palifermin-treated group. A slight increase in collagen accumulation was observed in the muscularis mucosa (magnification, ×100; staining, hematoxylin-eosin and Masson’s trichrome).

In the S/P-treated group, regular, preserved layers of mucosal, submucosal and muscular structures were observed. In the muscularis mucosa, a slight increase in collagen accumulation was observed following staining with Masson’s Trichrome (Fig. 3).

Statistically significant differences with regard to submucosal collagen accumulation, muscularis mucosa and tunica muscularis injury were observed between the B group and the C group (P<0.05). Similarly, statistically significant differences in the three parameters were observed when comparing the S and S/P-treated groups with the B group (P<0.05). In addition, submucosal collagen accumulation increased (P=0.007), and injury to the tunica muscularis was present (P=0.041) in the S-treated group when compared with the S/P-treated group (Table III).

### Stenosis index

Parameters used to calculate the stenosis index are shown in Table III. Statistically significant differences were observed among all the groups (P<0.05). The stenosis indexes in the C and S groups were significantly lower when compared with the B group (P<0.05). In addition, the stenosis index in the S/P group was significantly lower compared with the steroid-treated group and the control group (P<0.05).

## Discussion

Ingestion of caustic materials is a serious problem in children. Ingested alkaline materials penetrate rapidly into the esophageal wall and cause liquefaction necrosis with destruction of the mucosal, submucosal and, in severe cases, muscular layers of the esophagus. Following ingestion of the caustic material, hemorrhage, thrombosis of the submucosal vessels and inflammation with tissue edema develop in the first 24 h. Thrombosis may further increase the damage of the caustic burn and result in local necrosis and gangrene, with inflammation extending through the muscle layers and causing perforation. Following the sub-acute phase, scar formation begins with fibroblastic proliferation in the third week. Stricture formation occurs via the synthesis, deposition and remodeling of collagen. Different treatment strategies target different phases of the injury. During the early period, the main aim is to prevent stricture formation (1).

The optimal management protocols for the treatment of severe damage following the ingestion of caustic substances remain controversial. The main aim of medical treatment is the inhibition of any inflammatory reaction or stricture formation caused by esophageal burning. It is hypothesized that stricture formation may be overcome by inhibiting fibroplasia and scarring. In numerous experimental and clinical studies, it has been demonstrated that a combination of steroids and antibiotics are effective for mild or moderate corrosive burns. However, the drugs were unable to satisfactorily prevent stricture formation (13). Previous experimental studies have indicated that the administration of miscellaneous agents, including heparin, penicillamine, β-aminopropionitrile, indomethacin, pentoxifylline, estradiol, antioxidants, epidermal growth factor and vitamin E, may exert protective effects during the early phase of burn injury (13–17). In addition to these agents, esophageal stenting during the early stages has been used without steroids in clinical and experimental studies (18,19).

KGF is a growth factor that induces the proliferation, differentiation and migration of epithelial cells. A number of studies have demonstrated that KGF also plays key roles in inflammation and repair processes, as well as exhibiting a cell protective effect. Thus, KGF was approved by the FDA for use in clinical settings for the treatment of oral mucositis and ulcers (8,20). Using a rat model of an esophageal ulcer, Baatar *et al* (21) demonstrated that local administration of palifermin significantly increased epithelial cell proliferation on the ulcer border and promoted ulcer healing (21). However, to the best of our knowledge, no previous studies have reported the use of palifermin for the treatment of corrosive esophageal burns.

Exogenous KGF treatment following various types of injury significantly increases re-epithelization and causes major stimulation during the repair process. This evidence indicates that KGF treatment may reduce or prevent the deep penetration of damage in the acute phase of corrosive damage by increasing the epithelization in the esophageal lumen. Considering that steroid treatment inhibits endogenous KGF expression in tissue injuries, it may be hypothesized that the steroidal effect on delayed healing partially occurs via this mechanism (22). However, steroid treatment does not significantly affect the expression of KGF receptors. Therefore, it was hypothesized that the addition of palifermin to the existing treatment regime involving steroids may prevent the negative effects of steroids. In addition, palifermin may aid steroids in the anti-inflammatory response by downregulating the expression of proinflammatory cytokines, including interferon-γ and tumor necrosis factor-α, and may contribute to a reduction in the damage that occurs during the acute phase of inflammation by upregulating the expression of detoxifying enzymes that exert a protective effect against free oxygen radicals (23–25).

The results of the present study indicate that the administration of palifermin decreases the inflammatory response and reduces esophageal damage, which are important considerations for the sequelae of alkaline burns in esophageal injury.

The early inflammatory response and mucosal necrosis in burn tissue are known to be major causes of stricture in corrosive esophageal burns. During the early phase, the inflammatory response leads to necrosis and the further destruction of the esophageal wall. Bacterial translocation occurring on the acutely injured esophageal wall causes a more severe clinical situation. Following initiation of the burn, early and fast re-epithelization of the esophageal lumen, as a result of palifermin treatment, may have a protective effect against late phase stricture development by preventing additional inflammatory processes, including potential reflux esophagitis, bacterial and fungal colonization and exposure to saliva and mucosal secretions.

In conclusion, the results of the present study demonstrate the efficacy of palifermin treatment as an adjunctive therapy to the conventional treatment involving steroids plus antibiotics in a corrosive esophageal burn model. Palifermin treatment was shown to significantly decrease the degree of fibrosis and ameliorate histopathological damage in an experimental caustic burn model. However, since the present study was the first to use palifermin in the treatment of corrosive esophageal burns, further detailed animal and/or clinical studies, with a larger sample size, are required to support the current results.

## Figures and Tables

**Table I:** Histopathological analysis criteria.

Histopathological parameters	Score
Submucosal collagen accumulation	
Absent	0
2-fold increase in muscularis mucosa thickness	+1
>2-fold increase in muscularis mucosa thickness	+2
Muscularis mucosa injury	
Absent	0
Present	+1
Tunica muscularis injury and collagen accumulation	
Absent	0
Mild (collagen accumulation around the smooth muscles)	+1
Prominent (collagen accumulation around the smooth muscles and translocation of smooth the muscles with collagen)	+2

**Table II:** Body weights of the animals in each group.

Group	Weight at day 1 (g)	Weight at day 21 (g)	Change (%)
Control	203.6±5.4	225.0±7.4	11.0↑
Burn	201.5±4.0	175.5±4.6^a^	11.4↓
Steroid	202.3±4.7	215.6±6.3	10.6↑
Steroid + palifermin	203.1±4.2	222.1±6.2^b^	10.9↑

Values are presented as the mean ± standard deviation.

a P=0.012, vs. control group;

b P=0.012, vs. burn group.

**Table III:** Analysis of the stenosis index.

Group	n	Stenosis index
Control	8	0.32±0.05
Burn	6	0.95±0.07^a^
Steroid	8	0.57±0.06^a^,^b^
Steroid + palifermin	8	0.41±0.03^a^,^b^,^c^

Values are expressed as the mean ± standard deviation for all the groups.

a P<0.05, vs. control group;

b P<0.05, vs. burn group;

c P<0.05, vs. steroid group.

**Table IV:** Histological grading of the injuries among the study groups.

Group	Submucosal collagen accumulation	Muscularis mucosa injury	Tunica muscularis injury collagen accumulation
Control	0	0	0
Burn	1.5	1.0	1.5
Steroid	1.0	0.4^a^	0.8^a^
Steroid + palifermin	0.3^b^,^c^	0.2^b^	0.2^b^,^c^

a (P<0.05) and

b (P<0.05) compared with the burn group;

c (P,0.05)compared with the steroid group.
